# Facile synthesis of Al-doped NiO nanosheet arrays for high-performance supercapacitors

**DOI:** 10.1098/rsos.180842

**Published:** 2018-11-28

**Authors:** Jinping Chen, Xianyun Peng, Lida Song, Lihan Zhang, Xijun Liu, Jun Luo

**Affiliations:** Center for Electron Microscopy and Tianjin Key Lab of Advanced Functional Porous Materials, Institute for New Energy Materials and Low-Carbon Technologies, School of Materials Science and Engineering, Tianjin University of Technology, Tianjin 300384, People's Republic of China

**Keywords:** Al doping, NiO nanosheet arrays, electrochemical property, asymmetric supercapacitor

## Abstract

Electrode material design is the key to the development of asymmetric supercapacitors with high electrochemical performances and stability. In this work, Al-doped NiO nanosheet arrays were synthesized using a facile hydrothermal method followed by a calcination process, and the synthesized arrays exhibited a superior pseudocapacitive performance, including a favourable specific capacitance of 2253 ± 105 F g^−1^ at a current density of 1 A g^−1^, larger than that of an undoped NiO electrode (1538 ± 80 F g^−1^). More importantly, the arrays showed a high-rate capability (75% capacitance retention at 20 A g^−1^) and a high cycling stability (approx. 99% maintained after 5000 cycles). The above efficient capacitive performance benefits from the large electrochemically active area and enhanced conductivity of the arrays. Furthermore, an assembled asymmetric supercapacitor based on the Al-doped NiO arrays and N-doped multiwalled carbon nanotube ones delivered a high specific capacitance of 192 ± 23 F g^−1^ at 0.4 A g^−1^ with a high-energy density of 215 ± 15 Wh kg^−1^ and power density of 21.6 kW kg^−1^. Additionally, the asymmetric device exhibited a durable cyclic stability (approx. 100% retention after 5000 cycles). This work with the proposed doping method will be beneficial to the construction of high-performance supercapacitor systems.

## 1. Introduction

With the sharply increasing environmental pollution and the energy crisis over the past decades, the development of renewable energy storage systems has been paid increasing attention [1,2], of which supercapacitors have attracted considerable interest due to their quick charge/discharge rate, long lifespan, ultrahigh power density and reliability [3–6]. Generally, supercapacitors can be categorized into electrochemical double-layer supercapacitors and pseudocapacitors, which correspond to the ‘ion adsorption’ and the ‘fast surface redox reactions' electron storage mechanisms, respectively.

At present, some low-cost transition metal oxide materials, such as NiO, Co_3_O_4_, MnO_2_ and their composites, have attracted much interest mainly due to the inherent variable oxidation state of reversible electrochemistry [7–15]. Among them, NiO is a promising pseudocapacitor electrode candidate because of its high theoretical specific capacitance (2584 F g^−1^), robust chemical/thermal stability, environmentally benign nature, low cost and a wide range of preparation strategies [16–20]. However, its huge volumetric change during the charge/discharge process, intrinsically low electronic conductivity and low specific surface area limit its commercial applications [19].

In order to overcome these drawbacks, numerous efforts have been made, such as surface modification, synthesis of nanoscale NiO [21], incorporation with carbon materials [22,23] and doping [24]. The introduction of dopants (e.g. Al^3+^ and Co^2+^) has been suggested as a promising strategy for enhancing the electrochemical activity of TMO electrodes [24]. For example, Ren *et al.* show that Hollow NiO@Co_3_O_4_ nanofibres have the highest specific capacitances (788 F g^−1^ at the current density of 5 mA cm^−2^ and high cyclic performance [25]). Li *et al*. prepared an Al-doped MnO_2_ electrode material with high conductivity through high-energy ball milling [26]. Thereafter, Hu *et al*. synthesized α-MnO_2_ doped with Al using a hydrothermal method, and the Al-doped α-MnO_2_ achieved good electrochemical behaviour with a higher specific capacitance and perfect cycling stability [27]. Fan *et al.* reported a strategy synthesis of NiO foams on nickel foam substrates, which can greatly improve the areal performance of battery-type supercapacitors [28]. Al-doped β-Ni(OH)_2_ layered double hydroxide electrodes exhibited a specific capacitance of 2122.6 F g^−1^ at 1 A g^−1^ and long cycle life [22]. Meanwhile, it is well known that an ideal electrode for a high-performance supercapacitor should possess a large specific surface area [29,30]. Among various nanostructures, a nanosheet array can offer a large specific surface area, enlarging the contact surface between electrodes and electrolytes and facilitating the charge and mass transfers during electrochemical reactions. In addition, the direct construction of nanosheet arrays on a highly conductive substrate can avoid the use of polymer binders and conductive additives, which substantially reduces dead volumes in electrode materials.

Herein, Al-doped NiO nanosheet arrays grown on the surface of nickel foams (NFs) were synthesized by a facile hydrothermal method and an annealing treatment process. Compared with undoped NiO nanoarrays, the as-prepared Al-doped NiO nanosheets exhibited a higher capacitance (1679 ± 46 F g^−1^ at 20 A g^−1^), namely approximately 50–168% improvement in the specific capacitance. Besides, it showed a good cycling behaviour (1% capacitance loss after 5000 cycles) and a wonderful rate capability (remaining approx. 75% from 1 to 20 A g^−1^). Furthermore, a fabricated asymmetric supercapacitor (ASC) based on Al-doped NiO nanosheet arrays and N-doped multiwalled carbon nanotubes (MWCNTs) achieved superior energy density and power density of 215 ± 15 Wh kg^−1^ and 21.6 kW kg^−1^, respectively, as well as a robust cycling stability (approx. 100% retention after 5000 cycles). These findings demonstrate that the nanosheets can function as binder-free supercapacitor electrodes with improved capacitive performances, which can address the challenge for the application of NiO in electrochemical energy storage devices and beyond.

## 2. Experimental details

### 2.1. Materials synthesis

N-doped MWCNTs were purchased from the XFNANO Materials Company, and all other reagents (analytical grade) were from Sinopharm Chemical Reagent Co., Ltd. They were used as received without further purification.

A clear solution was prepared by mixing 1 mmol Ni(NO_3_)_2_·6H_2_O) and 1 mmol Al(NO_3_)_3_·9H_2_O in 50 ml deionized water at room temperature. Afterwards, 12.5 mmol CON_2_H_4_ (urea) and 10 mmol NH_4_F were introduced into the above solution under continuous stirring, leading to a solution denoted as Solution 1. Solution 1 was then transferred to a 50 ml Teflon-lined stainless-steel autoclave. An NF was carefully cleaned by immersing it in a 1 M HCl solution, deionized water and then ethanol, respectively, of which each immersion was accompanied by an ultrasound bath for 5 min. Then, the NF was immersed in Solution 1 in the autoclave, which was sealed, heated to 140°C and maintained for 10 h. Afterwards, a thin film on the NF was obtained and washed with deionized water and ethanol several times. The thin film was then dried at 80°C for 6 h. Finally, an Al-doped NiO nanosheet array was obtained by calcining the film with the NF at 250°C for 3 h. Additionally, Al-doped NiO arrays with different Al contents were prepared by adjusting the amount of Al(NO_3_)_3_·9H_2_O. Undoped NiO arrays without Al doping were prepared using the same conditions described above but without Al(NO_3_)_3_·9H_2_O [31].

### 2.2. Characterization

X-ray powder diffraction (XRD) was carried out at a scan rate of 10° per minute on an X-ray diffractometer (Rigaku D/max 2500). The scan angle, 2*θ*, ranges from 20° to 90°. The morphology of the as-synthesized samples was characterized using a field-emission scanning electron microscope (SEM) (Zeiss Supra 55) that was operated at 20 kV. The structures and compositions of the samples were characterized using high-resolution transmission electron microscopy (HRTEM; JEM 2100) with a field-emission gun at 200 kV and X-ray photoelectron spectroscopy (XPS) (Perkin-Elmer PHI 5600 spectrophotometer). The operating conditions of XPS were kept at 187.85 eV and 250.0 W. The ICP-MS measurements were conducted with an ICP Perkin-Elmer Optima 3000 DV. The electrical conductivities were measured using a four-probe conductivity meter (Loresta-GX MCP-T700).

### 2.3. Electrochemical measurements

The electrochemical measurements were performed in a three-electrode glass cell at room temperature (RT). A sample, namely an as-synthesized thin film on an NF (1 × 1 cm^2^), was directly used as the working electrode. The counter and the reference electrodes were a Pt foil and a saturated calomel electrode, respectively. A freshly prepared aqueous solution of 1 M KOH was used as the electrolyte. Electrochemical impedance spectroscopy (EIS) measurements were performed at open-circuit potential in the frequency range of 0.01 kHz to 100 Hz at an amplitude of 5 mV. In order to improve the accuracy, three identical samples were tested for each measurement.

The specific capacitance (*C*_sp_) of a sample was calculated according to the following equation (2.1):

2.1 $$C_{\mathrm{sp}}=\frac{I\Delta t}{m\Delta V},$$

where *I* (A) stands for the discharge current, Δ*t* (s) means the discharge time, *m* (g) is the total mass of electrochemically active materials and Δ*V* (V) signifies the discharge potential window.

To acquire the electrochemically active surface areas (ECSA) of the working electrode, their roughness factor (*R*_f_) should be obtained first according to the equation: ECSA = *R*_f_*S*, where *S* was generally equal to the geometric area of electrode (in this work, *S* = 1.0 cm^−2^). The *R*_f_ was determined by the relation *R*_f_ = *C*_dl_/20 µF cm^−2^ based on the double-layer capacitance (*C*_dl_) of a smooth metal surface (20 µF cm^−2^), where *C*_dl_ could be acquired by cyclic voltammetry (CV) measurement under the potential windows of 0.2−0.4 V. The scan rates were 5, 10, 15, 20 and 25 mV s^−1^. The *C*_dl_ was estimated by plotting *j*_a_ − *j*_c_ at 0.3 V (where *j*_c_ and *j*_a_ are the cathodic and anodic current densities, respectively) against the scan rate, where the slope was twice that of *C*_dl_. All electrochemical measurements were performed on an electrochemical workstation (CHI 670D).

### 2.4. Fabrication of an asymmetric supercapacitor device

In order to assemble an Al-doped NiO//N-doped MWCNT ASC device, an N-doped MWCNT electrode based on an NF was fabricated through a slurry-casting process. The slurry, containing N-doped MWCNTs, acetylene black and poly(tetrafluoroethylene) suspension (60 wt.%) binder at a weight ratio of 8 : 1 : 1, was coated on the NF and subsequently dried at 70°C for 24 h. The ASC was assembled based on an Al-doped NiO array directly as an electrode, the N-doped MWCNT electrode and 1 M KOH, which were used as the positive electrode, the negative electrode and the electrolyte, respectively. The cell balance was obtained by adjusting the electrode mass ratio of anode/cathode at 0.2, according to the result of the three-electrodes electrochemical measurement. The specific capacitance (*C*_device_), energy density (*E*), and power density (*P*) were calculated on the basis of equations (2.2), (2.3) and (2.4), respectively.

2.2 $$C_{\mathrm{device}}=\frac{I\Delta t}{M\Delta V},$$

2.3 $$E=\frac{C_{\mathrm{device}}\Delta V^{2}}{2}$$

2.4 $$\;\mathrm{and}\;P=\frac{E}{\Delta t},$$

where *I* (A) stands for the discharge current, Δ*t* (s) means the discharge time, *M* (g) means the total mass of both positive and negative electrodes, and Δ*V* (V) signifies the discharge potential window.

## 3. Results and discussion

**Figure 1.**
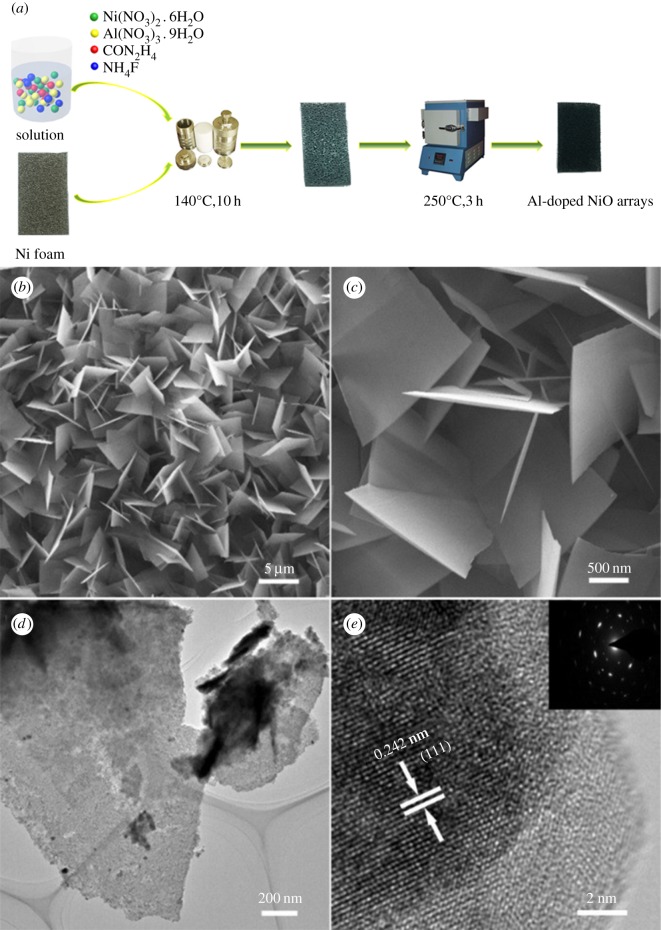
(*a*) Methods for preparing Al-doped NiO catalyst (*b*,*c*) low- and high-magnification SEM images of Al-doped NiO nanosheet arrays. (*d*,*e*) TEM and HRTEM images of the nanosheets. The inset in (*e*) is an SAED pattern corresponding to the HRTEM image.

Al-doped NiO nanosheets arrays were synthesized by a hydrothermal reaction and heat treatment. The preparation process was displayed in figure 1*a*. As shown in figure 1*b*, Al-doped NiO nanosheets grew vertically and interconnected with one another on the surface of NF to form a particular three-dimensional nanosheet array. Such structural and morphological characteristics are beneficial to making electrolytes accessible to the nanosheet surface during electrochemical processes. Figure 1*c* shows that the average length, width and thickness of individual nanosheets with a paper-like shape are about 2 µm, 5 µm and 40–50 nm, respectively. In addition, the interconnected feature of these nanosheets enables a large specific surface area, which increases the contact area of electrodes and electrolytes and then favours the full use of the active materials. Further, the TEM and the HRTEM images (figure 1*d*,*e*) show that the nanosheets are porous and composed of nanocrystals with a size of 10–20 nm. The selected area electron diffraction (SAED) pattern in figure 1*d* has the shape of concentric rings, which is the typical SAED pattern of polycrystalline materials. Therefore, Al-doped NiO nanosheet arrays show the nature of polycrystal, and the HRTEM image clearly reveals that the nanosheets have a distinct set of lattice fringes with an interplanar spacing of 0.242 nm, in a good agreement with the (111) plane of NiO. In addition, from the results of energy dispersive spectrometer mapping of Al-doped NiO nanosheet arrays (electronic supplementary material, figure S1), it can be seen that element Al, Ni, O is uniformly distributed in the nanosheet structure, which indirectly indicates that the composition of Al-doped NiO nanosheet arrays is uniform.

**Figure 2.**
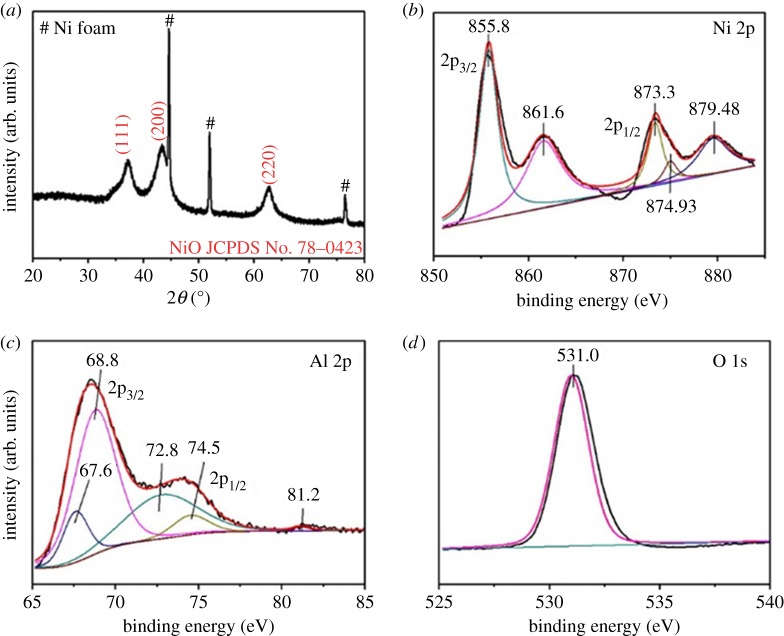
(*a*) XRD pattern of an Al-doped NiO nanosheet array. (*b*) Ni 2p XPS, (*c*) Al 2p XPS and (*d*) O 1 s XPS of an array. JCPDS is Joint Committee Powder Diffraction Standards.

The diffraction peaks (figure 2*a*) of the Al-doped NiO arrays can be unambiguously assigned to face-centred cubic phase NiO (No. 78-0423 of JCPDS) except that the diffraction peaks marked with # are ascribed to NF. Three typical peaks are clearly observed at 37.1°, 43.2° and 62.9°, which are indexed to the (111), (200) and (220) crystal planes of NiO, respectively. The XRD pattern of an undoped NiO array is shown in electronic supplementary material, figure S2, which is the same as figure 2*a*. Thus, the Al-doped NiO samples have the same crystal structure as the undoped NiO, indicating that the Al dopant atoms did not disturb the NiO lattice.

Figure 2*b*–*d* gives the XPS analysis of an Al-doped NiO nanosheet array. The Ni 2p spectrum (figure 2*b*) shows a characteristic fitting peak at 855.8 eV, which is consistent with Ni 2p_3/2_. The peaks at 873.3 eV and 874.93 eV correspond to Ni 2p_1/2_ in the NiO phase [32]. Besides, two shake-up satellite peaks (expressed as ‘Sat.’) are also observed at 861.6 eV and 879.48 eV [33]. These characteristic peaks of Ni 2p clearly indicate that Ni was present as Ni^2+^, in accordance with the reported [34]. Moreover, as shown in figure 2*c*, it can be seen that four observable peaks located at 67.6 eV (Al 2p_3/2_), 68.8 eV (Al 2p_3/2_), 72.8 eV (Al 2p_1/2_) and 74.5 eV (Al 2p_1/2_), which clearly indicate the presence of Al^3+^ in the NiO product [35]. The high-resolution spectrum of the O 1 s (figure 2*d*) displays a strong metal-oxygen peak at 531.0 eV, which is indicative of the lattice oxygen associated with Al-O and Ni-O [36]. Obviously, it can be confirmed that this array contains Al^3+^ and Ni^2+^. Its Al/Ni atomic ratio was determined by inductively coupled plasma mass spectrometry (ICP-MS) as 6.6/93.4, suggesting that its Al content is 3.3 at%.

**Figure 3.**
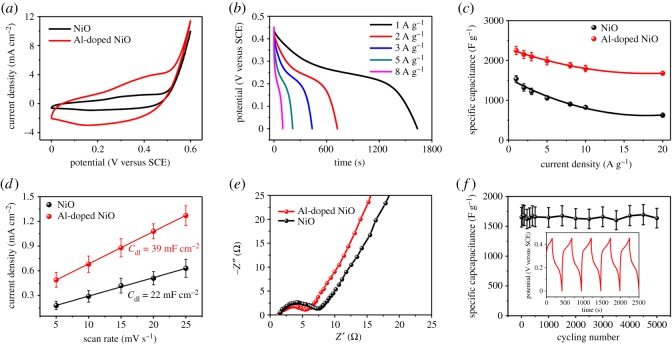
Electrochemical characterization of undoped and Al-doped NiO nanosheet arrays: (*a*) CV curves of the electrodes at a scan rate of 5 mV s^−1^. (*b*) Galvanostatic discharge curves of the Al-doped NiO nanosheet array at various discharge current densities (see the electronic supplementary material, figure S3 for undoped NiO). (*c*) Specific capacitance versus different current densities. (*d*) Charging current densities plotted against scan rates, in which *C*_dl_ is double-layer capacitance. (*e*) EIS spectra. (*f*) Average specific capacitance versus cycle number at a galvanostatic charge/discharge current density of 20 A g^−1^, in which the inset shows the last five cycles of the charge–discharge curves.

To evaluate the electrochemical performance of the samples as electrodes in electrochemical capacitors, the CV curves of undoped and Al-doped NiO electrodes were measured at a scan rate of 5 mV s^−1^ with a potential window ranging from 0 to 0.6 V in a 1 M KOH solution. The results are shown in figure 3*a*. As expected, the Al-doped NiO electrode exhibits a much higher capacitive current density than that of the undoped NiO. Its curve contains a pair of redox peaks at 0.45 V and 0.17 V, which is related to the redox behaviour of the electrode material (NiO + OH^−^ → NiOOH + H_2_O + e^−^). These results indicate the typical Faradaic characteristics of the as-prepared electrode materials.

The galvanostatic charge/discharge curves of the undoped and the Al-doped NiO electrodes were obtained within the voltage range of 0–0.45 V at various current densities (figure 3*b* and electronic supplementary material, figure S3). The discharge capacitance of the Al-doped NiO electrode is much higher than that of the undoped NiO electrode at a discharge current density of 1 A g^−1^, which is in accordance with the afore-mentioned CV results. Electronic supplementary material, figure S4 presents the specific capacitance of Al-doped NiO electrodes with various Al content at a discharge current density of 1 A g^−1^. Clearly, it can be seen that the optimal Al content in the Al-doped NiO samples is 3.3 at% with a maximum specific capacitance at a discharge current density of 1 A g^−1^. The presence of Al causes the electrochemical performances of NiO electrode towards a higher value. It is possible that Al-doped NiO-based film forms β-phase Ni(OH)_2_ firstly which is stable in KOH resolution. Al-doping could prevent the crystallization of the electrode, which exhibited much better electrochemical and electrochromic properties [37]. However, too much Al will be reacted with KOH resolution primarily, leading to the decline of the specific capacitance. Thus, there is an optimal doping content of Al to obtain the maximum specific capacitance. Furthermore, figure 3*c* shows the specific capacitances of the samples corresponding to the discharge curves at various discharge current densities. The Al-doped NiO electrode exhibits impressive discharge capacitances (2253 ± 105 F g^−1^ and 1679 ± 46 F g^−1^ at 1 A g^−1^ and 20 A g^−1^, respectively). Obviously, the Al-doped NiO electrode shows an improved rate capability compared to the undoped NiO electrode.

The enhanced specific capacitance of the Al-doped NiO electrode might originate from a larger electrochemically active area when compared with the undoped counterpart. To verify this viewpoint, the *C*_dl_ values of these products were determined. Figure 3*d* reveals that the *C*_dl_ for Al-doped NiO was 39 mF cm^−2^, larger than that of undoped NiO, confirming that the Al-doping increased the electrochemically active area.

On the other hand, the Al doping can enhance the conductivity of the electrodes, which was confirmed by the EIS analysis in electronic supplementary material, figure S5. The Nyquist plot comprised a quasi-semicircle at the higher frequency region and a linear part at the lower frequency region. The semicircles of the EIS spectra correspond to the Faradic reactions, and their diameters represent the interfacial charge-transfer impedances. The linear parts correspond to the Warburg impedance (*W*), which is described as a diffusive impedance of the OH^−^ ion within an electrode. The equivalent circuit of measuring impedance data is drawn in electronic supplementary material, figure S5b, which involves the internal resistance (*R*_s_), double-layer capacitance (*C*_dl_) and Faradic charge-transfer resistance (*R*_ct_), Warburg diffusion element (*Z*_w_) and pseudocapacitance (*C*_F_) [38]. Equations (2.1) and (2.2) express *Z*, which is the total resistance of the equivalent circuit in electronic supplementary material, figure S5a, where *j* is the imaginary unit, *ω* is the angular frequency (Hz) and *W* is the Warburg parameter in units of Ω s^−1/2^. At sufficiently high frequencies, the overall impedance can be reduced to equation (2.3), corresponding to the track of Nyquist plot intersecting axes at *R*_s_ and *R*_s_ + *R*_ct_ [39].

3.1 $$Z=R_{s}+\frac{1}{\;j_{\omega }C_{dI}+1/(R_{\mathrm{ct}}+Z_{w})}-j\frac{1}{\omega C_{F}},$$

3.2 $$Z_{w}=\frac{W}{\sqrt{\;j_{\omega }}}$$

3.3 $$\;\mathrm{and}\;Z=R_{s}+\frac{1}{\;j\omega C_{\mathrm{dI}}+R_{\mathrm{ct}}}.$$

Electronic supplementary material, figure S5a shows the Nyquist plots of the experimental impedance data and fitting results of the Al-doped NiO nanosheet arrays and the undoped NiO. It can be seen clearly from the electronic supplementary material, figure S5a, that the doped electrode exhibited lower pseudo charge-transfer resistance (*R*_ct_) than the undoped one, indicating improved electronic and ionic conductivities [40]. In addition, the measured square resistance of the Al-doped NiO electrode was 9.25 × 10^5^ Ω sq^−1^, smaller than that of the undoped NiO (4.16 × 10^7^ Ω sq^−1^), which is consistent with the EIS results. For the two electrodes, the slopes of the straight lines at low frequency are almost the same, indicating a nearly equal value of electrolyte diffusion impedance.

Furthermore, a long-term cycling stability is an important criterion for practical supercapacitor applications. Figure 3*f* presents the cycling property of the Al-doped NiO electrode, which exhibited an inconspicuous decay specific capacitance after 5000 cycles at a current density of 20 A g^−1^. The high stability indicates that the charging/discharging processes do not induce a structural change of the doped electrode as expected for faradaic redox reactions. In addition, the representative charge/discharge curves remain in a good symmetry, indicating that the Al-doped NiO electrode has good capacitive characteristics (inset of figure 3*f*).

**Figure 4.**
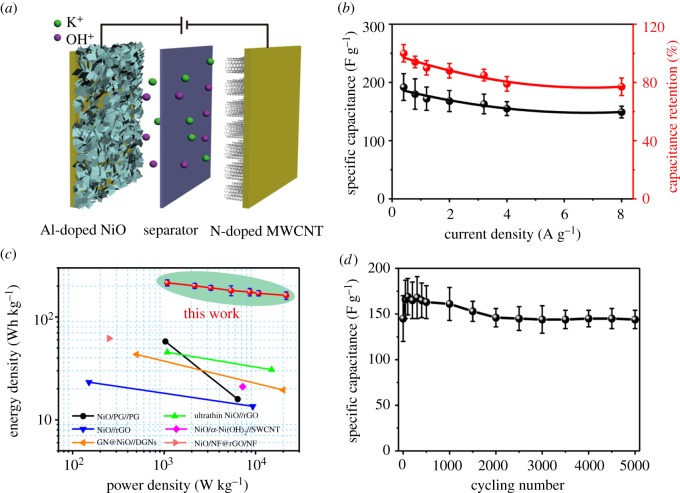
(*a*) Schematic illustration of an Al-doped NiO//N-doped MWCNT ASC. (*b*) Specific capacitance and capacitance retention at different current densities. (*c*) Ragone plot of the estimated energy and power densities at different current densities for the hybrid device. (*d*) Cycling stability of the hybrid device.

To further evaluate the electrochemical capacitor performance of the Al-doped NiO nanosheet arrays for practical application, an ASC was assembled based on an Al-doped NiO array and N-doped MWCNTs (electronic supplementary material, figure S6), which were used as positive and negative electrodes, respectively. The schematic illustration of the Al-doped NiO//N-doped MWCNT ASC device was displayed in figure 4*a*. Electronic supplementary material, figure S7 shows its galvanostatic discharge curves at various current densities between 0 and 1.5 V. The calculated specific capacitance (based on the total mass of the active materials of the two electrodes) of the ASC and the capacitance retention are shown in electronic supplementary material, table S1, respectively. Thus, these results imply a high-rate capability (figure 4*b*). Based on the discharge curves, the energy and the power densities for the Al-doped NiO//N-doped MWCNT electrochemical capacitor at various current densities are displayed in the Ragone plot (figure 4*c*). Clearly, the present device exhibited a maximum energy density of 215 ± 15 Wh kg^−1^ at a power density of 1.08 kW kg^−1^ and an energy density of 162 ± 14 Wh kg^−1^ at the highest power density of 21.6 kW kg^−1^, which further demonstrate the practical applicability of the fabricated Al-doped NiO//N-doped MWCNT ASC device. Moreover, the cycling stability of the hybrid device was measured at a current density of 20 A g^−1^ for 5000 charge/discharge cycles. As shown in figure 4*d*, the results clearly show 100% retention after 5000 cycles, indicating a high cycling stability of the electrochemical device.

## 4. Conclusion

In summary, Al-doped NiO nanosheet arrays were fabricated using a facile and effective strategy for constructing an ASC. They exhibited superior discharge capacitance (2253 ± 105 F g^−1^ at 1 A g^−1^), rate capability (approx. 75% capacitance retention at 20 A g^−1^) and cycling stability (approx. 99% retention after 5000 cycles), much improved compared to those of undoped NiO. The enhanced electrochemical properties are attributed to the nanosheet array feature and the doping-induced improvements in conductivity. Moreover, an ASC was constructed by an Al-doped NiO nanosheet array and nitrogen-doped MWCNTs, and it showed an outstanding energy density (215 ± 15 Wh kg^−1^), superior power density (21.6 kW kg^−1^) and an impressive cycling performance (approx. 100% capacitance retention after 5000 cycles). This work can help develop novel electrode material for high-performance pseudocapacitors.

## Supplementary Material

Additional figures

## Data accessibility

The datasets supporting this article have been uploaded as part of the electronic supplementary material.
