# Supplementary material for: Facile synthesis of Al-doped NiO nanosheet arrays for high-performance supercapacitors
Source: R Soc Open Sci. 2018 Nov 28;5(11):180842. doi: 10.1098/rsos.180842 (PMC6281943; doi:10.1098/rsos.180842)
Supplement: Additional figures [file rsos180842supp1.docx]

Electronic Supplementary Material for

**Facile synthesis of Al-doped NiO nanosheet arrays for high-performance supercapacitors**

Jinping Chen, Xianyun Peng, Lida Song, Lihan Zhang, Xijun Liu* and Jun Luo

Center for Electron Microscopy, Tianjin Key Lab of Advanced Functional Porous Materials, Institute for New Energy Materials and Low-Carbon Technologies, School of Materials Science and Engineering, Tianjin University of Technology, Tianjin 300384, China

* Corresponding authors.

E-mail addresses: xjliu@tjut.edu.cn (X.J. Liu)

**
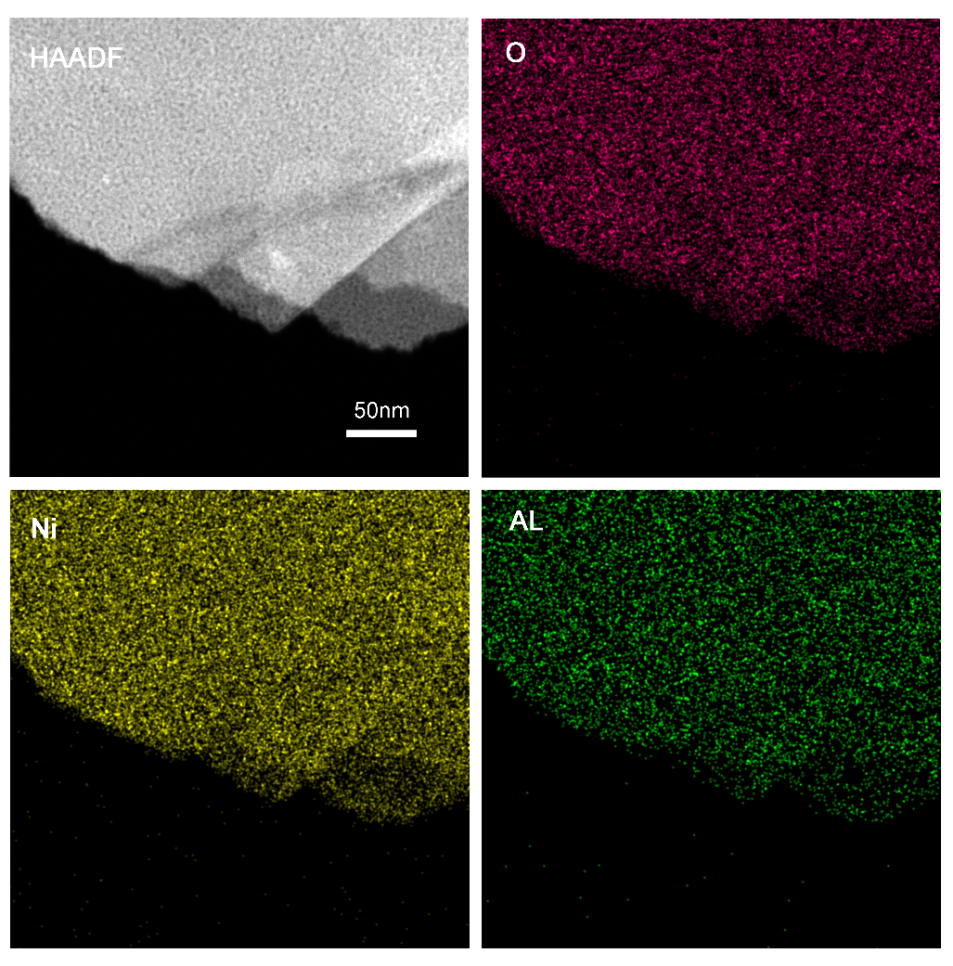
**

**Fig. S1.** HAADF images and EDS elemental mapping of Al, Ni, and O of Al-doped NiO nanosheet arrays.


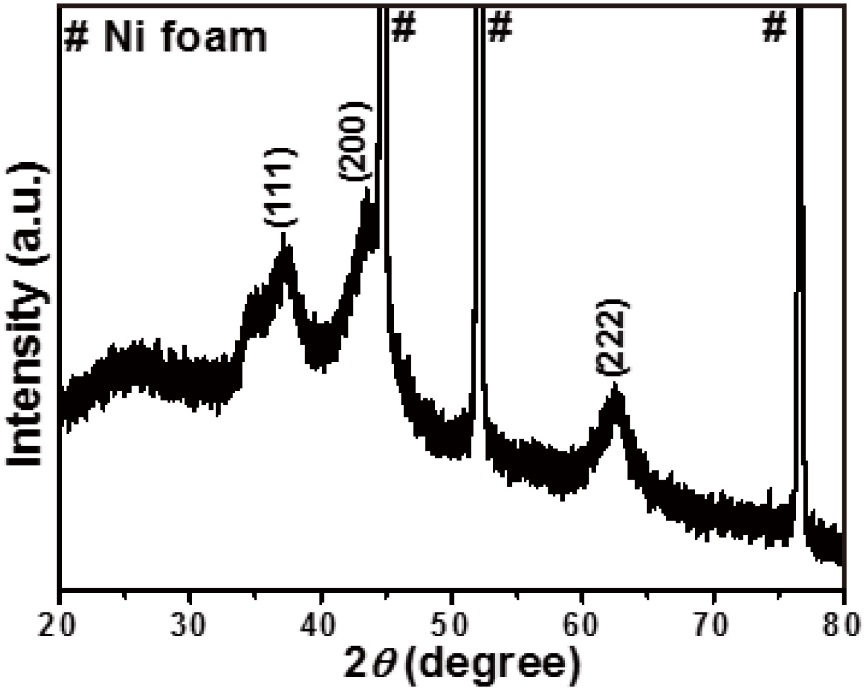


**Fig. S2.** XRD patterns of pure NiO.


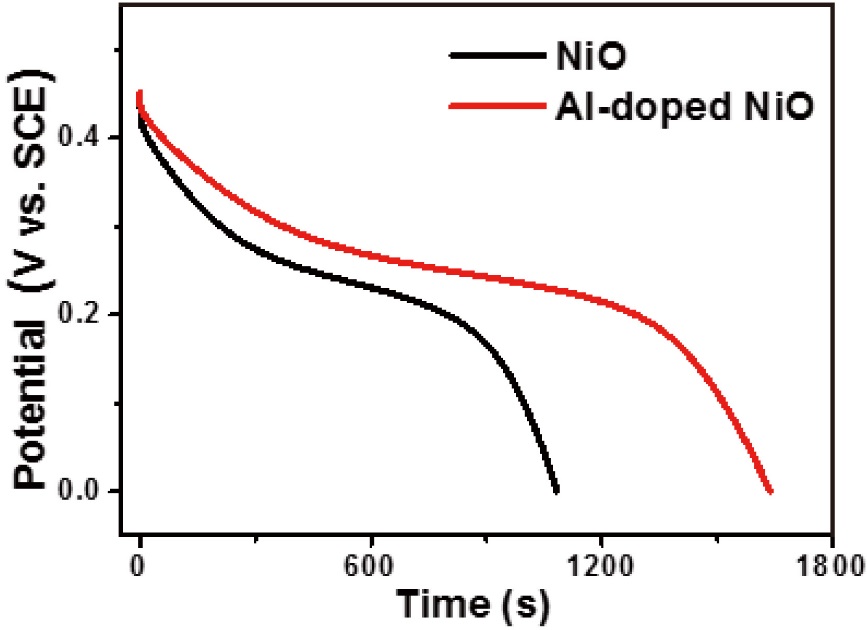


**Fig. S3.** Galvanostatic discharge curves of NiO and Al-doped NiO electrodes at a discharge current density of 1 A g^-1^.

**
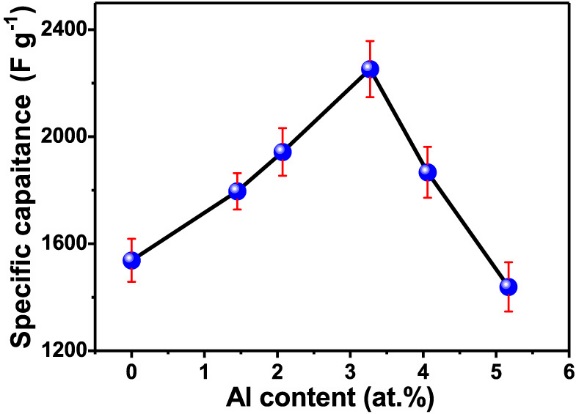
**

**Fig. S4.** The specific capacitance of Al-doped NiO electrode with various Al content at a discharge current density of 1 A g^-1^.

**
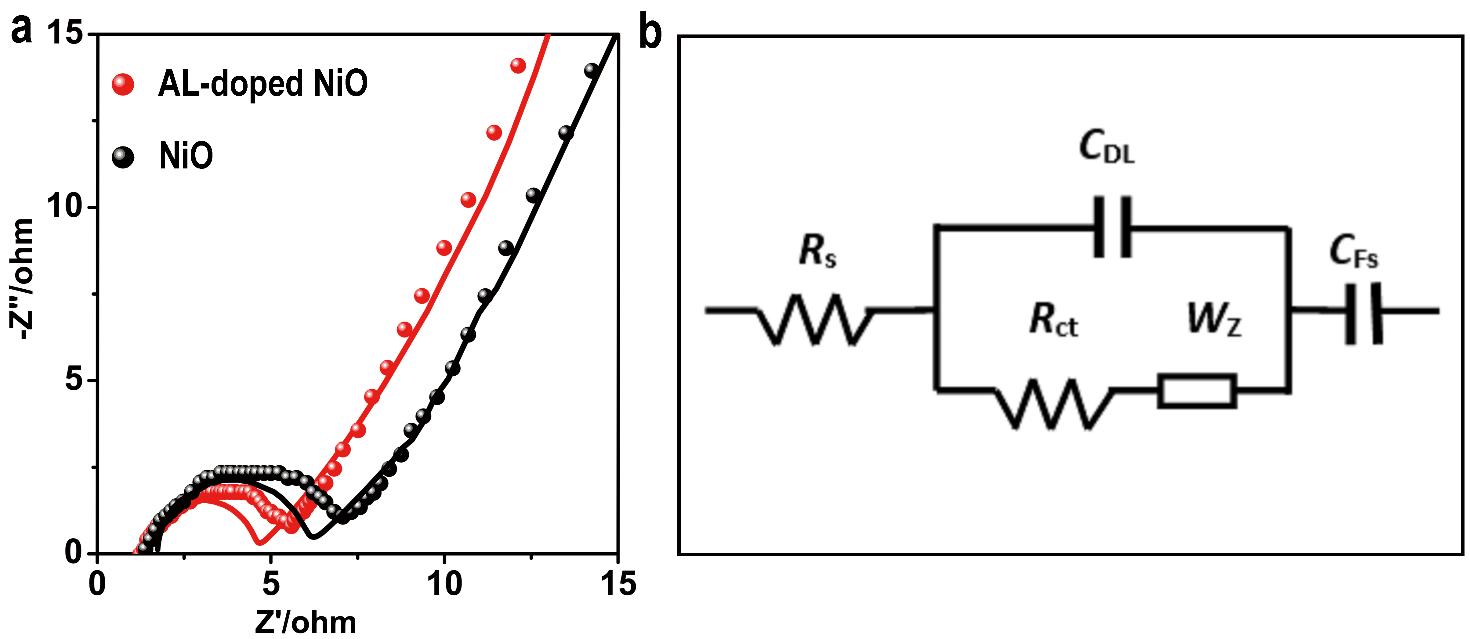
**

**Fig. S5.** Nyquist plots of the experimental impedance data (scattering dot) and fitting results (solid line) of the Al-doped NiO nanosheet arrays and the undoped NiO).

**
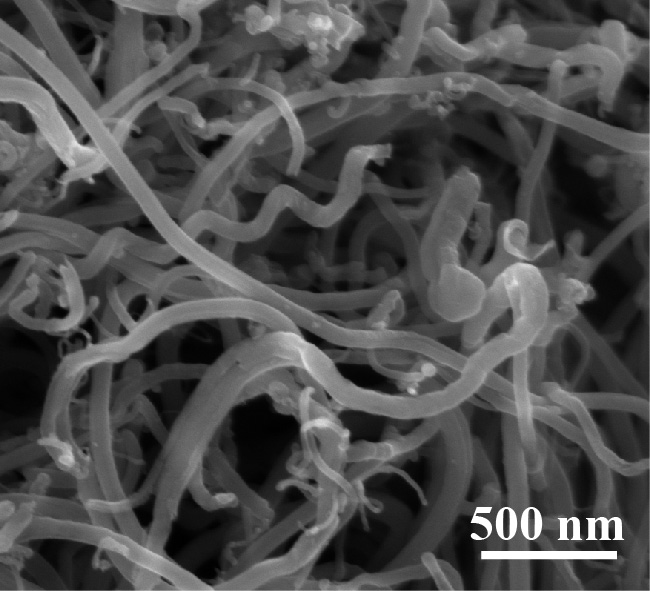
**

**Fig. S6.** SEM image of multiwalled carbon nanotube.


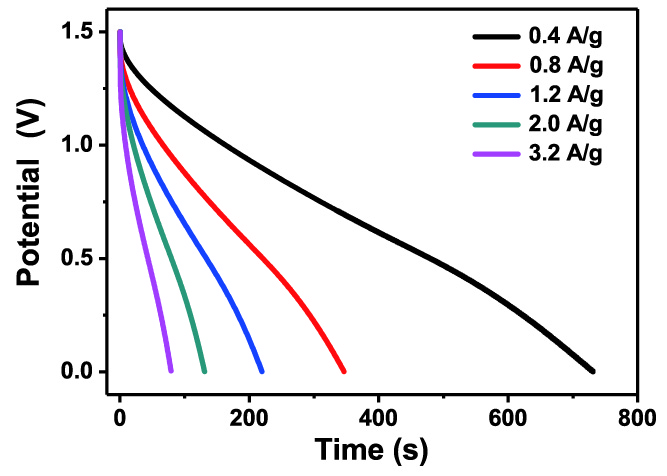


**Fig. S7.** The galvanostatic discharge curves of the Al-doped NiO//N-doped MWCNT ASC at different discharge densities.

**Table S1** Specific capacitance and capacitance retention at different current densities.

| **Current density**  **(A g^-1^)** | **Specific capaitance**  **(F g^-1^)** | **Capaitance retention**  **(%)** |
| --- | --- | --- |
| 0.4 | 192 ± 23 | 100 ± 6 |
| 0.8 | 180 ± 26 | 94 ± 4 |
| 1.2 | 172 ± 20 | 90 ± 5 |
| 2 | 168 ± 18 | 88 ± 5 |
| 3.2 | 163 ± 17 | 85 ± 4 |
| 4 | 155 ± 12 | 79 ± 5 |
| 8 | 149 ± 10 | 77 ± 6 |
